# Editorial: The connections of immune metabolic mechanisms with aging-related diseases

**DOI:** 10.3389/fcell.2023.1295264

**Published:** 2023-09-27

**Authors:** Ozlem Tufanli, Mevlut Citir, Changjun Yin, Emiel P. C. Van der Vorst, Ismail Cimen

**Affiliations:** ^1^ Altos Labs, Bay Area Institute of Science, Redwood City, CA, United States; ^2^ Institute of Precision Medicine, The First Affiliated Hospital of Sun Yat-sen University, Guangzhou, China; ^3^ Institute for Cardiovascular Prevention (IPEK), Ludwig-Maximilians-University Munich, Munich, Germany; ^4^ Institute for Molecular Cardiovascular Research (IMCAR), RWTH Aachen University, Aachen, Germany; ^5^ Aachen-Maastricht Institute for CardioRenal Disease (AMICARE), RWTH Aachen University, Aachen, Germany; ^6^ Interdisciplinary Center for Clinical Research (IZKF), RWTH Aachen University, Aachen, Germany

**Keywords:** immune cells, metabolic mechanisms, immunometabolism, aging-related diseases, metabolites

## Introduction

Aging is a complex phenomenon that causes the accumulation of cellular damage, leading to an increased incidence of major age-related diseases such as cancer, neurodegenerative, cardiovascular diseases (CVD), and immune system diseases (Guo et al., 2022). The main features of aging are known to be metabolic alterations at cellular and systemic levels (Bevilacqua et al., 2023). Metabolic pathways of glucose, lipid, and amino acids have considerable importance in immune cell functions, such as differentiation, migration, and immune responses (Pearce and Pearce, 2013). Immune cells through metabolic pathways produce molecules, metabolites, and energy necessary for the events related with a new cellular state (Riffelmacher et al., 2018). Metabolites are indispensable factors for the immune system involved in both metabolic circuits and signaling cascades. In mammals, the endocrine and nervous systems as well as the microbiota release various metabolites such as neurotransmitters, hormones, bile acids, short-chain fatty acids, and indoles, all of which impact the biology and behavior of immune cells, largely by acting on specific receptors (Zhang et al., 2022).

Among these metabolites, short-chain fatty acids (SCFAs) play an important role in aging related diseases such as cardiovascular diseases (CAD). In this Research Topic, Guo et al. focus on the specific effects of butyrate in human aortic endothelial cells (HAOECs). Butyrate is a gut microbiota-derived metabolite that is associated with vascular integrity and development of CAD. The authors elucidated a mechanism through which butyrate is acting on vascular integrity. They found that butyrate treatment induced phosphorylation of several tyrosine sites on vascular endothelial cadherin (VEC) in HAOECs, with the largest effect seen on tyrosine-731. By performing chemical inhibition and siRNA knockdown experiments, they further demonstrated that the butyrate-induced phosphorylation of VEC was mediated by c-Src kinase and (free fatty acid receptor 2)/FFAR3 (free fatty acid receptor 3) receptors. The butyrate-induced VEC phosphorylation was ultimately associated with remodeling of junctional VEC and increased endothelial cell permeability. Hence, the study sheds light on the potential impacts of butyrate on CAD Guo et al..

Additionally, caloric restriction (CR) might also affect the aging process by favorably influencing human health, and it is of critical importance for defining the underlying signaling mechanisms of aging (Kim et al., 2020). A recent study has reported that CR imprints immune cells for gaining better control of *Mycobacterium tuberculosis* (MTB) growth, activating proteolytic processes, being more energetic, and remaining protected against oxidative damage. Moreover, immune cells acquire the capacity to control immunometabolic alterations triggered by MTB infection (Palma et al., 2021). Mechanisms that prolong longevity via CR modulation of autophagic function or epigenetic modifications have also gradually been investigated. In the context of aging, Zhai et al. summarized CR-induced autophagy and epigenetic modifications on both DNA and histones to explore the molecular mechanism of CR to delay aging and age-related diseases, and the interactions between autophagy and epigenetic modifications. Furthermore, they described several calorie-restriction mimetics (CRMs) that exert similar actions on the regulation of autophagy and the epigenome. Precise regulation of autophagy and understanding its molecular interactions with epigenetic modifications may potentially reveal new rejuvenation approaches that can be further explored in the future Zhai et al.

Another process that influences immunometabolism of both innate and adaptive immune cells, and immune responses is autophagy and autophagy-related processes. Santovito et al. have compiled the evidence supporting the role of autophagy in cardiac homeostasis, aging, and cardio immunological response to cardiac injury. As reported in this review, age related dysregulation of autophagy related signaling pathways enhances susceptibility to aging-associated cardiac dysfunction. In addition to this evidence, many studies proved that manipulation of autophagy through several mechanisms such as, ubiquitous overexpression of *Atg5,* transgenic overexpression of Parkin (mitophagy related gene) reduced aging associated cardiac damage and increased the lifespan in mice. Although strong evidence highlights the potential benefits of autophagy manipulation to revert age-induced cardiac dysfunction, the role of autophagy in ischemia-reperfusion injury is still not entirely understood. Furthermore, autophagy has a crucial role in orchestrating immune cell function and their response to cardiac injury and ischemia. In the final part of this review, the authors summarized recent advances in targeting autophagy in cardiac diseases. Studies suggest that the level of autophagy should be in an optimal zone to be able to get benefit from autophagy targeting drugs. Autophagy levels that are outside of this therapeutic window (higher or lower) can pose a deleterious effect on health. However, there is still a great medical need to identify such proper therapeutical window especially in diseased conditions and for well-tolerated pro-autophagic drugs Santovito et al.

Both innate and adaptive immune cells aggregate to the adventitia and sense atherosclerotic plaques. A more recent study demonstrated that the adventitial neuroimmune cardiovascular interfaces (NICIs) forms a biologically active anatomically discernible structure in which the immune system interacts with both the diseased artery and the nervous system in a tripartite complex tissue network (Mohanta et al., 2022). Mohanta et al. have provided original contributions to this Research Topic by discussing the neuroimmune cardiovascular interfaces in atherosclerosis. Early studies showed that the majority of immune cells accumulated in the adventitia region of late-stage atherosclerosis and large B cell aggregates observed in advanced diseased artery segments. The authors termed these aggregates *artery tertiary lymphoid organs* (ATLOs) and through a series of imaging and functional experiments, they showed that ATLOs harbor multiple B cell subtypes, plasma cells and separate T cell areas. Axon endings are also highly enriched in these areas, showing sympathetic nervous system restructuring. This expanded axonal network increases with aging. They proposed a direct crosstalk between the diseased arterial wall and both the immune and the nervous system in tripartite rather that bidirectional interactions. As reported in this review further studies should be directed to elucidate more detailed morphology and function of NICIs and characterize the axon tips in the adventitia Mohanta et al.

In summary, this Research Topic highlights several immune-metabolic mechanisms, like metabolites, caloric restriction, autophagy and neuroimmune cardiovascular interfaces that play a key role in aging and aging-related diseases, providing and discussing novel findings fueling this field of research.
